# Towards Zero Zinc Oxide: Feeding Strategies to Manage Post-Weaning Diarrhea in Piglets

**DOI:** 10.3390/ani11030642

**Published:** 2021-02-28

**Authors:** Andrea Bonetti, Benedetta Tugnoli, Andrea Piva, Ester Grilli

**Affiliations:** 1 Dipartimento di Scienze Mediche Veterinarie (DIMEVET), Università di Bologna, via Tolara di Sopra 50, 40064 Ozzano dell’Emilia (BO), Italy; andrea.bonetti15@unibo.it (A.B.); andrea.piva@unibo.it (A.P.); 2 Vetagro S.p.A., via Porro 2, 42124 Reggio Emilia, Italy; benedetta.tugnoli@vetagro.com; 3 Vetagro Inc., 116 W. Jackson Blvd., Suite #320, Chicago, IL 60604, USA

**Keywords:** zinc oxide, post-weaning diarrhea, *Escherichia coli* F4, K88, ETEC, piglet, pig nutrition, weaning

## Abstract

**Simple Summary:**

Zinc oxide (ZnO) supplementation at pharmacological doses in post-weaning piglets is a consolidated practice that allows efficient control of post-weaning diarrhea (PWD), a condition exacerbated by *Escherichia coli* F4 (K88) infections. Far from being completely elucidated, the multifactorial ZnO mechanism of action is in all likelihood exerted at the gastrointestinal level. However, increasing environmental concerns are arising from prolonged ZnO use. This article reviews the utilization of ZnO in piglets, the biological rationale behind its powerful activity, and the emerging threats that are leading towards a significant reduction in its use. Finally, a wide analysis of the strengths and weaknesses of innovative alternative strategies to manage PWD at the nutritional level is given.

**Abstract:**

Zinc oxide (ZnO) at pharmacological doses is extensively employed in the pig industry as an effective tool to manage post-weaning diarrhea (PWD), a condition that causes huge economic losses because of its impact on the most pivotal phase of a piglet’s production cycle. In a multifactorial way, ZnO exerts a variety of positive effects along the entire gastrointestinal tract by targeting intestinal architecture, digestive secretions, antioxidant systems, and immune cells. ZnO also has a moderate antibacterial effect against *Escherichia coli* F4 (K88), the main causative agent of PWD. However, the environmental impact of ZnO and new emerging threats are posing serious questions to the sustainability of its extensive utilization. To work towards a future free from pharmacological ZnO, novel nutritional approaches are necessary, and many strategies have been investigated. This review article provides a comprehensive framework for ZnO utilization and its broad mode of action. Moreover, all the risks related to pharmacological ZnO levels are presented; we focus on European institutions’ decisions subsequently. The identification of a novel, complete solution against PWD should be accompanied by the adoption of holistic strategies, thereby combining good management practices to feeding approaches capable of mitigating *Escherichia coli* F4 (K88) infections and/or lowering ZnO utilization. Promising results can be obtained by adjusting diet composition or employing organic acids, natural identical compounds, polyphenol-rich extracts, prebiotics, and probiotics.

## 1. Introduction

Pigs are one of the most important livestock species, being the world’s primary meat resource for human nutrition along with poultry [1]. According to OECD-FAO 2019–2028 projections, pork will remain a pivotal nutritional source of animal protein in both developed and developing countries [2]. To face the expected continuous consumption, which will mirror the population growth and the increasing access of developing countries to newer meat varieties, attention to production cycles will be more and more necessary, to identify critical phases and recognize all the essential strategies to improve animal performance.

Concerning pigs, the most pivotal stage of their breeding is weaning, a sudden and extremely stressful moment [3]. In the modern pig industry, weaning generally occurs 21–28 days after birth [4,5]. Several aspects contribute to the consideration of weaning as a challenging state, ranging from psychosocial factors to dietary changes and morpho-functional alterations along the digestive tract [6,7,8]. The switch from a liquid to a solid diet damages intestinal villi, with an impairment in intestinal absorption and secretion of digestive enzymes, thereby leading to a reduced growth rate, starvation periods, and severe weaning anorexia [9,10]. Noteworthy effects also occur in the stomach, where the secretion of HCl is not sufficient to ensure proteolysis and protection against pathogens, which finally reach the hindgut [10,11,12]. Moreover, because of the sudden lack of passive immunity provided through maternal milk and the underdevelopment of the piglet’s immune system at weaning, acute immunological changes occur and an inflammatory status is established [10,13].

All these interconnected circumstances exert detrimental effects on the overall health status of animals, providing the ideal environment for the onset of post-weaning diarrhea (PWD), one of the most economically-relevant diseases in pig husbandry due to the costs of therapies, slower growth, and increased mortality [14]. The main causative agent is enterotoxigenic *Escherichia coli* (ETEC), hence the second name of PWD: post-weaning colibacillosis. The most common strain associated with PWD occurrence in piglets worldwide is *Escherichia coli* F4 (*E. coli* F4, also known as *E. coli* K88), which infects animals via the oral route [15]. Bacterial ancillary plasmids are responsible for the pathogenicity of these strains: they harbor not only antimicrobial resistance genes, but also sequences encoding fimbrial adhesins, essential for ETEC pathogenicity and capable of interacting with enterocytes’ receptors [16]. However, the onset of diarrhea is primarily due to the heat-labile (LT) and the two heat-stable (STa and STb) toxins that *E. coli* K88 can secrete to target intestinal epithelial cells. Although triggering different pathways, all the toxins disrupt tight-junctions, activate massive luminal secretions of electrolytes and water, and finally bring about diarrhea [17,18].

For a long time, zinc oxide (ZnO) has been widely used at high doses as an effective means to prevent diarrhea and control *E. coli* F4 infections. However, starting from June 2022, medicinal doses of ZnO will no longer be authorized in the European Union, so novel strategies to manage PWD are urgently needed. Starting from this general framework, the aim of this review is to investigate the rationale behind the large-scale utilization of ZnO in post-weaning piglets, its precise mechanism of action, and the reasons that are causing researchers, industries, and political institutions to reduce its employment at high levels. Additionally, the present work provides an overview of all the main in-feed alternatives to the use of medicinal levels of ZnO and strategies to control *E. coli* F4; their strengthens and limitations are described as well.

## 2. Zinc Oxide

Zinc (Zn) is one of the most important trace elements in animal nutrition because it ensures the activity of several enzymes involved in cellular signaling, digestion, cellular respiration, and nucleic acid metabolism [19,20]. Zinc deficiency has been correlated with growth retardation, lower appetite, worsening of feed conversion rate, and skin complications [21].

In diets, Zn can be supplemented in many different forms. The addition of Zn in the form of ZnO represents the commonly recommended way to prevent and reduce the incidence of PWD. The administration of ZnO in piglets usually lasts 14 days, starting immediately after weaning, the time during which its highest beneficial effects are observed [22,23]. In vivo studies proved that—to exert its positive action—ZnO inclusion in feeds should reach a concentration greater than 1000 ppm, with an optimum of 2500 ppm of Zn (equivalent to approximately 3100 ppm of ZnO), a dose generally defined as “pharmacological” [24]. Davin and colleagues proved that weaning causes a Zn deficiency (low plasma Zn concentration) that can be counteracted by pharmacological ZnO [25]. As demonstrated in rats, increasing levels of dietary ZnO, although ensuring an incremental absorption of Zn, do not implicate a surge in Zn retention, thereby proving that pharmacological doses of ZnO overcome physiological needs and that beneficial effects do not strictly depend on the fulfilment of nutritional Zn requirements [26].

The supplementation of ZnO not only results in better fecal scores at weaning [27,28] and a lower appearance of PWD symptoms and mortality [29], but also improves piglets’ growth performance [22,30], digestion [31], and feed intake [32]. Hahn and Baker demonstrated that the best result in daily weight gain was obtained for plasma Zn levels between 1.5 and 3 mg/L, an ideal concentration reached by supplementing exactly 3000 ppm of ZnO [33]. Despite increasing plasma Zn concentration, other Zn forms provided at the same level did not report significant enhancements in growth parameters, thereby suggesting that Zn’s efficacy is likely independent from its effective absorption, but it should exert a primary action within the gut [33].

### 2.1. Mechanism of Action of ZnO

**Figure 1:**
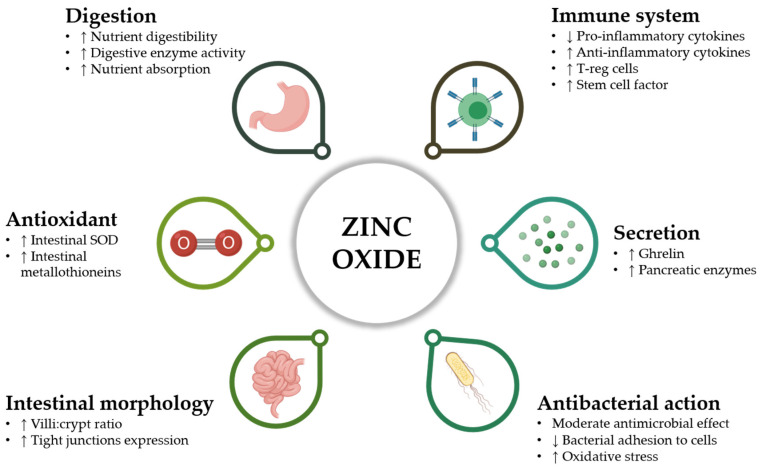
Beneficial effects and mechanisms of action of zinc oxide in post-weaning piglets. For each parameter, the up arrow “↑” means an increase, while the down arrow “↓” identifies a reduction.

The precise mechanism of action of ZnO against PWD has not yet been fully elucidated. However, many studies have hypothesized and demonstrated several beneficial effects of ZnO on different targets, as summarized in Figure 1. Rather than the fulfilment of Zn daily nutritional requirements, the main mechanism of action of ZnO seems to be related to a substantial enhancement in nutrient absorption and intestinal morphology [34]. Indeed, further studies confirmed that the supplementation of pharmacological ZnO improves performance by ensuring higher villi:crypt ratio, greater occludin protein levels, and a considerable proliferative effect on enterocytes [35,36]. The increase in tight-junction expression is a clear marker of ZnO ability to reduce intestinal permeability in a very delicate phase for piglets [37], with a marked influence on the appearance of diarrheic symptoms.

The positive effect of ZnO on intestinal mucosa can be also related to its antioxidant properties. Zn is an essential ion for the catalytic action of superoxide dismutase (SOD), whose level is increased in the intestine of ZnO-supplemented piglets [36], and for the expression of metallothioneins, which possess an antioxidant action for their metal-binding capacity [38,39] and that may play a role in cell growth and development [40]. For what concerns bacteria, ZnO causes the onset of oxidative stress in microbial cells: in aqueous solutions, high doses of ZnO generate reactive oxygen species that, thanks to their direct action on cell walls, can damage bacterial cells [39,41]. This mechanism of action could partially explain the antimicrobial activity that many Zn forms exert mostly at concentrations equivalent to the medicinal doses used in animal diets [42,43].

Despite this potential mechanism, the actual effect of ZnO against *E. coli* F4, the main causative agent of PWD, is moderate. Indeed, ZnO antimicrobial activity seems to be mainly addressed against Gram-positive rather than Gram-negative bacteria [42], as also confirmed by microbiota analysis, with ZnO supplemented animals reporting higher coliforms and enterococci but lower lactic acid bacteria [44]. Furthermore, pig diets added with 2500 ppm of ZnO show no differences in the number of fecal coliforms if compared to controls [45]. Considering that the low diarrhea occurrence in ZnO-supplemented piglets is recurrently associated with a persistent fecal excretion of *E. coli*, it is realistic that mechanisms of action different from a direct antimicrobial activity do exist for ZnO [46].

An in vitro study from Roselli et al. confirmed the limited direct antimicrobial activity of ZnO against *E. coli* K88, emphasizing instead the capacity of ZnO to protect cultured enterocytes from ETEC-induced damages. Indeed, it is demonstrated that ZnO inhibits bacterial adhesion to cells, thereby preventing the disruption of the intestinal tight-junctions, as proved by the higher trans-epithelial electrical resistance (TEER) values for infected cells when supplemented with ZnO [47].

Cytokines are major inflammation players that influence the tight-junction functioning of epithelial cells [48]. During in vitro infections of intestinal epithelial cells with *E. coli* F4, ZnO significantly reduced the expression of several pro-inflammatory cytokines and improved the transcription of the anti-inflammatory cytokine TGF-β [47], together with a significant downregulation of genes related to the innate immune response [49]. The same modulatory effect on intestinal inflammation was demonstrated by many in vivo studies, which reported lower IFN-γ, TNF-α, and IL-6 in ZnO-supplemented piglets [35,36,50].

Not only is ZnO capable to modulate cytokines expression, but also it is an essential trace element with a key action on the entire immune system, for both its development and its correct function [50]. Deficiency of Zn brings to a significant decrease of macrophage capacity to perform phagocytosis [51] and to kill parasites [52]. Moreover, Zn seems fundamental to enhance the killing activity of natural-killer lymphocytes [53]. A disturbed Zn homeostasis has been correlated with an increased risk for infections, due to an impaired balance of T helper cells and to an increased apoptosis of immature T cell precursors [54], therefore proving the key role of Zn for T helper cell differentiation [55]. In weaned piglets, pharmacological doses of ZnO showed the ability to increase the number of regulatory T cells, which are pivotal for modulating the immune response and maintaining homeostasis [56]. A study from Ou and colleagues highlighted the role of pharmacological doses of ZnO in reducing the expression of stem cell factor in the small intestine of weanling pigs, an effect that could not only repress maturation and proliferation of intestinal mucosal mast cells, but also decrease their release of histamine, a key molecule for the pathogenesis of diarrhea and one of the main players for the onset of an inflammatory status [57].

Researchers proved that pharmacological supplementation of ZnO also participates to the stimulation of ghrelin secretion at the stomach level of young pigs. Ghrelin action promotes the release of insulin-like growth factor 1 (IGF-1) and cholecystokinin (CCK) by the liver and the GI tract, respectively, thereby enhancing muscle protein synthesis, cell proliferation, and feed intake [58,59]. Moreover, Hedemann and colleagues verified that high dietary levels of ZnO in weaned pigs increase the activity of several pancreatic digestive enzymes such as carboxypeptidases, trypsin, and chymotrypsin [60], guaranteeing a higher nutrient digestibility. All these multiple effects can together contribute to the systematic performance improvement observed in weaning piglets fed with medicinal ZnO doses.

### 2.2. Risks Related to Pharmacological Levels of ZnO

**Figure 2:**
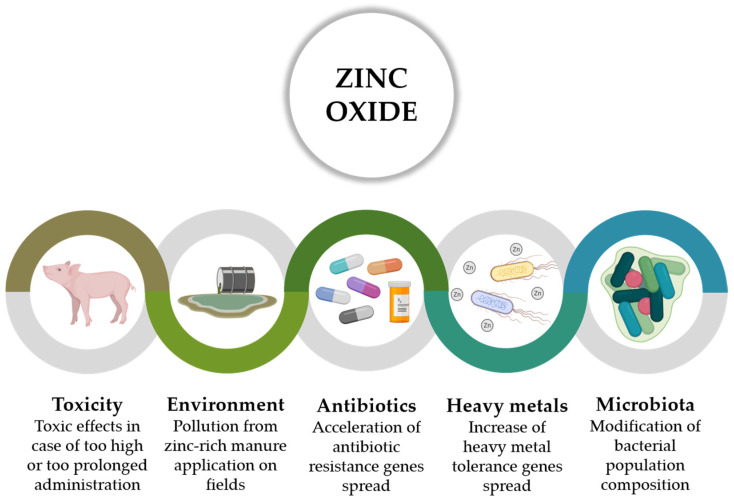
Risks related to pharmacological zinc oxide utilization in post-weaning piglets.

The large and prolonged employment of ZnO at pharmacological levels in pig farming has raised several concerns, as depicted in Figure 2. Too high or too much protracted ZnO supplementations lead to the loss of ZnO benefits with the potential onset of toxic effects [22,61,62], because of the excessive accumulation of Zn in animal tissues such as kidney, liver, and pancreas [63,64], which can experience Zn overload [62].

The main issue concerning ZnO is related to the considerable risks for the environment, deriving from the application of Zn-rich manure to land: manure coming from facilities employing pharmacological doses of ZnO is definitely abundant in Zn, considering that animals excrete all the excess of Zn that exceeds physiological requirements [22,65,66]. Due to the non-volatile and non-degradable physicochemical properties of Zn, the long-term continuous spread of manure on crops can progressively increase its concentration into soils and groundwaters, finally reaching dangerous levels for plant and animal life [67,68]. Bak et al. demonstrated that, over the period 1986–2014, the application of Zn-rich pig slurry led to a greater soil Zn concentration of 2–5%, with an average increase of 24% over the last sampling period (1998–2014), concluding that there might be significant risks for aquatic species because of Zn leaching from fertilized fields to groundwaters [69]. Although risk mitigation measures are more and more implemented, such as manure dilution and ensuring distance to surface waters, the European Medicines Agency (EMA) 2017 report about veterinary medicinal products containing ZnO concluded that these precautions only represent a delay for the unavoidable Zn accumulation in the environment [23].

Several studies proved that ZnO pharmacological supplementation in piglets might also contribute to the acquisition and spread of antibiotic resistance genes [70]. Slifierz et al. showed that therapeutic doses of ZnO can increase the persistence and prevalence of methicillin-resistant *Staphylococcus aureus* in weaning piglets, probably due to the colocalization of Zn and methicillin resistance genes [71]. The diffusion of resistance genes amongst *E. coli* inside the intestine of piglets weaned with high ZnO levels was also observed [72], together with a considerable increase of multi-resistant *E. coli* strains in feces, digesta, and colonic mucosa samples [73,74]. Piglets supplemented with high ZnO doses show a tendency into the selection of more heavy metal tolerant *E. coli* isolates such as strains harboring the *czrC* gene [75,76], thereby jeopardizing any potential antimicrobial activity of Zn.

The impact of ZnO on the intestinal microbiota is controversial. While some data only suggest minor or transient modifications to hindgut bacterial compositions [77,78,79], other studies reveal remarkable effects on porcine microbial populations [80]. It was demonstrated that pharmacological doses of ZnO can significantly increase the count of Enterobacteriaceae in piglets [81]. Moreover, ZnO can influence microbiota composition, by reducing *Lactobacillus* species and increasing *Clostridiales* and *Enterobacteriales* diversity [82,83]. The modulatory activity of ZnO on commensal microbiota probably resembles the activity of growth-promoting antibiotics: the suppression of Gram-positive species, without affecting Gram-negative strains, brings to lower bacterial activity and ATP concentration in the gut of piglets, which makes more energy available for the host [44], at the cost of losing, though, beneficial commensal bacteria.

Given all the risks linked to Zn, the European Food Safety Agency (EFSA) is constantly monitoring pollution levels and proposed a significant reduction in the use of Zn in feeds [84]. Moreover, to reduce the environmental and antimicrobial-resistance threats derived from the accumulation of Zn from medicated feeds, the EMA concluded that the benefit-risk balance for the utilization of medicinal ZnO containing products in pig breeding had a negative outcome. This resolution was dictated by the severe hazards for the environment, that took priority over the prevention of post-weaning diarrhea in piglets. The decision, issued on 26th June 2017, imposes a complete elimination of ZnO at medicinal levels in piglet feed, enforcing a five-year timeframe to phase these products out of the market [23]. No laws or restrictions are actually in force in other countries such as the United States, but breeders are always welcoming any new alternative strategy to manage PWD and *E. coli* F4 infections.

Since the European Commission has supported the EMA decision towards a future free from pharmacological levels of ZnO by 2022 [85], an urgent need for ZnO-reducing alternatives is arising. Indeed, following the EC regulation 1334/2003 and 2016/1095, ZnO will be only utilizable as a feed additive with the legal limit of 150 ppm of total Zn in complete feed [86,87]. This dose unlikely exerts the same effects of pharmacological levels of ZnO, so non-traditional approaches against PWD are urgently needed.

### 2.3. Why ZnO?

As an ion, Zn is contained inside a great number of compounds, some of which are of primary interest to fulfill Zn dietary requirements in animal nutrition. For this reason, many Zn-containing molecules have been tested as potential substitutes for pharmacological doses of ZnO in piglets, with the aim to supplement them at concentrations lower than the medicinal ones.

Together with ZnO, the most used source of Zn in animal husbandry is zinc sulphate. The reason why this alternative inorganic form is used relies on the higher bioavailability of Zn when bound to the sulphate group [88,89,90]. The investigation of the potential in vitro growth-inhibiting activity of Zn sulphate against a panel of intestinal pathogens (i.e., *Salmonella* spp., *Enterobacter* spp., *Escherichia coli*, *Vibrio cholerae*) showed that it exerts an antimicrobial action between 1.2 and 1.8 mg/mL, thereby suggesting a possible antidiarrheal activity not only linked to physiological effects, but also to a mild antibacterial action [43]. However, to the best of our knowledge, no comparative studies between pharmacological levels of ZnO and Zn sulphate have been published so far; thus, no reports support Zn sulphate as an alternative to ZnO.

Zinc chloride is another Zn inorganic compound employed in animal nutrition. Although being less studied, its beneficial effects are probably linked to the inhibition of certain bacterial populations and the improvement of intestinal health [91]. One of the most diffused forms of Zn chloride is tetrabasic zinc chloride (TBZC), an additive manufactured through reactive crystallization processes, with the final production of a water-insoluble colorless salt. This form is preferable because of the higher purity, the excellent palatability, the reduced reactivity with other feed nutrients, and the better bioavailability compared to ZnO [92,93]. In piglets, supplementation of 1000–1500 mg/kg of Zn chloride in the TBZC form can enhance fecal score and growth parameters as equally as pharmacological ZnO [94,95,96], however without being able to drastically reduce Zn doses below the limits imposed by the European Commission.

Effective Zn sources can also be organic Zn forms [97]. At pharmacological levels, Zn from Zn-methionine or Zn-lysine can increase plasma Zn concentration much more than ZnO or other inorganic Zn forms [33,98,99]. Bouwhuis and colleagues verified that the inclusion of 500 ppm of Zn-methionine in post-weaning piglets did not result in improved fecal scores and intestinal architecture at the same extent of 3300 ppm of ZnO [100]. Other research groups proved that 250–500 ppm of various organic Zn forms could increase to a certain extent the growth parameters of weaning piglets, however without reaching the performance of pharmacological levels of ZnO [32,101,102]. These results further prove that the mechanism of action of ZnO against PWD is not likely related to Zn absorption, so any utilization of organic Zn forms to improve Zn bioavailability would not exert desirable positive effects on PWD symptoms.

As a whole, all the previously presented alternatives do support the idea that the activity of Zn in preventing PWD or controlling *E. coli* F4 infections is not merely related to the supply of Zn, but rather it is strictly dependent to its inclusion in the form of ZnO. Considering that other Zn forms do not represent feasible alternative ways to reduce pharmacological doses of ZnO under the limit established by the European authorities, several approaches were explored to maintain the ZnO molecule by minimizing its levels with the aim to decrease its environmental footprint. The rationale behind the reduction of ZnO doses lies in its solubility at acidic pH [103]: in the low pH gastric environment, the majority of ZnO is dissolved as Zn^2+^ and eventually absorbed, with a limited amount flowing into the small intestine, where all the beneficial effects of ZnO are exerted [104]. Moreover, it is likely that Zn^2+^ dissolved ions, once entered the intestine, could react with NaOH forming Zn(OH)_X_, an insoluble and inactive Zn form. If ZnO is industrially processed, such as miniaturized, incorporated in minerals, or microencapsulated, a higher dose can reach the small intestine without being transformed and lost along the gastric tract, thereby allowing a significant reduction of ZnO concentration in animal feed.

Thanks to the great strides of nanotechnologies, an attractive form of ZnO is represented by ZnO nanoparticles (nZnO). Nanoparticles of ZnO possess better chemical stability and reactivity because of their smaller size, that brings to an increased number of particles and surface area per unit mass [105,106]. Moreover, nZnO show an antimicrobial activity against pathogenic bacteria such as *E. coli*, *Salmonella*, *Staphylococcus*, and *Listeria* [107]. Their mechanism of action is related to nZnO enhanced capacity to permeate into bacterial cell membranes, causing oxidative damages and lipid peroxidation [108]. In 2019, a study by Pei et al. proved that dietary supplementation of 450 ppm of ZnO nanoparticles in piglets diets could exert similar effects to the common ZnO pharmacological dose of 3000 ppm, maintaining improvement in growth and intestinal morphology parameters, with a significant reduction of fecal Zn excretion [109]. Additional findings proved other positive effects such as fecal *E. coli* count reduction and intestinal tight-junctions expression improvement [110]. However, other research groups did not confirm the enhanced performance parameters, or reported contrasting results on diarrhea rate when pharmacological ZnO was replaced with 500–1200 ppm of nZnO [106,110,111]. Furthermore, all the available studies report the use of nZnO at doses far from the strict limits imposed by European authorities, thereby making it difficult to suggest nZnO as an effective low-dose alternative to medicinal ZnO levels.

Several carrier minerals have been employed to control the release of ZnO along the gastrointestinal tract. The supplementation of 600–900 ppm of zeolite-supported ZnO proved to be as efficacious as pharmacological doses of ZnO in enhancing growth performance, maintaining intestinal barrier function, and alleviating diarrheic symptoms of post-weaned piglets [28]. The same beneficial effects, together with a significant improvement of tight-junction protein expression, was reported for 500 ppm of ZnO incorporated inside diosmectite [27]. Moreover, piglets fed with 500 ppm of montmorillonite-ZnO hybrid reported—as equally as pharmacological doses of ZnO—a noteworthy enhancement in growth performance, lower diarrhea, improvement of intestinal morphology, and higher activity of pancreatic and intestinal digestive enzymes [31,112].

One of the most efficient ways to diminish ZnO concentration originates from the microencapsulation technology, which was shown to allow for a reduced amount of ZnO requested to maintain health and performance of piglets after weaning [113]. As firstly demonstrated by Grilli et al., 150 and 400 ppm of microencapsulated ZnO were as effective as the free pharmacological dose of 3000 ppm, with beneficial effects on intestinal morphology, cell proliferation, gut inflammatory cytokines and tight-junction protein expression, and production parameters [35]. Subsequent studies proved similar effects: coated ZnO doses ranging from 380 to 500 ppm exerted positive actions on diarrhea incidence, intestinal development, mucosal immune system, and nutrient digestibility [104,114]. Moreover, low doses of coated ZnO could enhance intestinal antioxidant capacity and reduce intestinal cell apoptotic index [115], together with a significant decrease in fecal Zn [116] and environmental pollution.

Although the above presented approaches look promising in reducing the ZnO bioactive concentration and in controlling its environmental impact, all the employed doses are far from complying with the requirements imposed by the European Commission of 150 ppm of total Zn in complete feed from 2022. While pursuing a future without pharmacological ZnO, novel cutting-edge approaches are currently being investigated as effective pioneering ZnO substitutes to control PWD and *E. coli* F4 infections.

## 3. Alternatives to ZnO

Many non-feed strategies have been investigated to control PWD and *E. coli* F4 infections. Poor environmental conditions are considered contributing factors to the establishment of PWD [14]. Sanitary aspects deeply affect the overall health status of piglets: a deterioration in husbandry hygiene levels can trigger a background inflammation status that may intensify other stressors [117], finally leading to depressed growth performance and diarrhea [118]. Moreover, considering that some animals are not susceptible to ETEC infections because of genetic reasons [119,120], the breeding selection of animals resistant to *E. coli* F4 was considered as a solution to erase PWD, but several difficulties occurred [121,122].

Even if non-feed approaches all represent noteworthy factors to PWD onset and environmental enterotoxigenic *E. coli* persistence [123], their analysis is beyond the scope of this review. Since the mechanism of action of ZnO is primarily related to a wide-spectrum action on the gastrointestinal tract of weaning piglets, only feed-related strategies different from pharmacological ZnO levels will be discussed. For each proposed alternative approach, only studies including a clear comparison with high ZnO doses and/or investigating novel methods to control *E. coli* F4—the main etiological agent of PWD—were considered.

A prospect of all the different strategies to PWD and *E. coli* F4 is reported in Table 1, which summarizes all the advantages and limitations of each analyzed approach.

### 3.1. Adjusting Diet Composition: Protein and Fiber

At weaning, the high buffering capacity of the solid feed and the low HCl production of the piglet stomach lead to low protein digestion [124]. For this reason, a large amount of undigested protein flows into the large intestine, where it is easily fermented by the host microbiota causing an overgrowth of proteolytic bacteria—such as pathogenic *E. coli*—and their toxic fermentation by-products [141,142]. Moreover, undigested proteins can facilitate the onset of osmotic diarrhea. An effective way to control the protein amount arriving into the hindgut is the reduction of the dietary crude protein (CP) level simultaneously to the supplementation of essential amino acids (AA) [143]. Low CP and AA supplemented diets proved useful to enhance fecal consistency and decrease PWD incidence even in *E. coli* F4 experimentally challenged piglets without the aid of pharmacological ZnO up to 14 days after weaning [125,144]. Researchers also showed that weaned pigs fed low CP diets (16% instead of 20% CP) can reduce up to 30% the daily treatments for diarrheic symptoms in the 6–15 kg phase [145]. Furthermore, a low CP diet (15.5% instead of 20% CP) helps in lowering IL-1, TNF-α, IL-2, IL-6 and increasing IL-10 and IL-13 concentrations in the colonic mucosa of weanling piglets, probably due to a reduction of the inflammation elicited by the ingestion of antinutritional factors and feed antigens usually occurring with common protein sources such as soybean meal [146].

However, pig performance typically declines when dietary CP percent is heavily reduced, so lower productivity should be taken into account when opting for this alternative strategy to phase out medicinal levels of ZnO [144]. Even if later additions of dietary high CP can counterbalance for the initial lower performance [145], some compensatory strategies are currently under investigation, such as management precautions and supplementation of both essential and branched-chain AA [146,147,148]. Overall, these insights show that dietary CP plays a role in the PWD onset and that its concentration should be carefully determined to avoid an easier *E. coli* F4 gut colonization at weaning.

If acting with CP can be risky for the maintenance of piglets performance, several authors suggested the possibility to use insoluble fibers to control PWD symptoms. Fibers improve intestinal health by promoting the establishment of a healthy bacterial community in the hindgut [149]. Furthermore, fiber improves intestinal morphology [150] and prevents diarrhea formation by decreasing *E. coli* counts in feces [128].

Fibers such as insoluble non-starch polysaccharides (iNSP) have been employed with success in several studies. Fernandes et al. demonstrated that the combination of fibers with low doses of ZnO (500–800 ppm) allowed the same growth performance of pharmacological ZnO in piglets experimentally challenged with *E. coli* F4 [127]. Furthermore, researchers proved how supplementation of fibers could help in control PWD by reducing *E. coli* F4 faecal counts, and by improving short-chain fatty acids (SCFA) levels in the digesta [126,151]. The mechanism of action of iNSP is related to their ability to diminish the adhesion of pathogens such as *E. coli* F4 to intestinal epithelial cells, as demonstrated by in vitro studies on IPEC-J2 cells [152,153], and decrease the retention time of digesta [116]. The two mechanisms likely play a synergistic role because an increased passage rate can lower the attachment of pathogens to the intestinal surface.

### 3.2. Organic Acids

Organic acids (OA) are one of the most employed groups of feed additives in pig nutrition. In their classic role as acidifiers, OA can reduce gastric pH of weanling piglets counteracting the buffering capacity of the diet, ensuring a proper digestion of dietary proteins. Moreover, gastric acidification is also valuable as an antimicrobial barrier [12,154]. In addition, OA are also widely used for their direct antimicrobial activity and their ability to improve the overall health status of pigs gastrointestinal tract [129,155]. Several studies proved the beneficial effects of lactic, propionic, citric, formic, and caprylic acids supplementation in post-weaning piglets, which ensure a reduction in intestinal harmful coliform bacteria [156], a modulation of the immune response [157,158], and an improvement in diet digestibility [159], laying the ground for better performance and reduced post-weaning diarrhea incidence [130]. Moreover, it was demonstrated that butyrate can improve growth parameters in post-weaning piglets, also thanks to its capacity to modulate intestinal inflammation and integrity [160,161,162]. An in vitro study by our research group highlighted how selected OA can reduce the expression of many *E. coli* K88 virulence-related genes, ranging from toxins secretion to motility and quorum sensing [163]. A few studies in piglets experimentally challenged with *E. coli* F4 demonstrated that mixtures of short chain fatty acids (SCFA) can decrease diarrhea severity, improve growth performance, and reduce fecal *E. coli* counts [130,164]. Moreover, it was also demonstrated that a complex of SCFA with capric acid (a medium chain fatty acid) can significantly alleviate the inflammatory response in piglets challenged with *E. coli* F4 [165], remarkably reducing TNF-α and IFN-γ plasma levels. These findings ascribe OA as powerful alternatives to manage PWD, even if further studies are required to fully understand the effectiveness of OA to completely replace pharmacological doses of Zn.

### 3.3. Essential Oils and Nature Identical Compounds

Following the complete ban of antibiotics as growth promoters in the European Union, essential oils (EO) have immediately gained attention as potential alternatives in animal diets. Their usefulness is related to their strong antimicrobial, antioxidant, and anti-inflammatory activity [131,132,133]. The main etiological agent of PWD, *E. coli* F4, proved to be susceptible to several EO, including cinnamon, clove, and thyme oils [166,167,168]. Many in vivo studies confirmed positive effects deriving from the supplementation of EO blends in weanling piglets, with a reduction in coliform proliferation [156], a lower fecal score and diarrhea appearance [169], an improvement in intestinal morphology and feed digestibility [170], and an increase in total antioxidant activity [171]. However, studies providing EO supplementation sometimes report contrasting results or inconclusive outcomes [172,173], probably due to different EO inclusions and compositions, which could be significantly influenced by factors such as soil composition, plant species, harvesting season, extraction method, and stability [168,174].

For these reasons, nature identical compounds (NIC)—pure chemicals reflecting active principles of EO—are more and more employed to overcome variabilities and potentiate EO effects [133]. Thymol, carvacrol, and eugenol are three of the most effective NIC against *E. coli* F4, as demonstrated by their in vitro antimicrobial activity [153,159,168]. Moreover, we previously demonstrated that these three monoterpenes significantly downregulate mRNA levels of *E. coli* K88 genes involved in toxins production, bacterial adhesion to enterocytes, motility, and quorum sensing [163]. As regards in vivo trials, feeding a blend of thymol, vanillin, and OA for two weeks in post-weaning piglets resulted in a modulation of several inflammatory markers and an enhancement in growth parameters, while in vitro experiments showed a reduction in tight-junction permeability [175]. Moreover, the same blend significantly lowered the *E. coli* shedding and tended to contribute to the reduction of Zn excretion in feces of weanling pigs supplemented with low doses of microencapsulated ZnO [113]. Therefore, these data suggest NIC as promising candidates to serve as effective ZnO alternatives to manage PWD and *E. coli* F4.

### 3.4. Polyphenol-Rich Extracts

Plant and fruit extracts—referred as natural extracts (NE)—are widely used in traditional medicine to treat various diseases, including gastrointestinal conditions such as diarrhea. Several studies proved the effectiveness of watery or alcoholic NE extracts against enteric pathogens, with polyphenols being the class of molecules with the highest activity [176,177]. Due to their accumulation into the hindgut, polyphenols such as resveratrol, quercetin, and phenolic acids have a broad antimicrobial activity against microorganisms such as *S. aureus*, *Campylobacter*, *Salmonella*, and *E. coli* [135,178].

The antidiarrhoeic activity of polyphenols is probably related to their capacity to bind proteins. Historically, this property has labelled them as antinutritional factors, but research is now exploring their usefulness against pathogenic *E. coli* [136]. Several NE, together with their polyphenolic content, were shown to be active against *E. coli* F4 pathogenicity at different levels, ranging from a moderate antimicrobial activity [179] to the inhibition of LT toxin and the repression of enterocytic ionic channels [134,180,181]. Thus, targeting virulence instead of bacterial growth and survival represents a novel and pioneering approach to the pathogenicity of microorganisms, with the key advantage to preserve host microflora from unwanted harmful modifications and reduce selective pressure against the development of bacterial resistance [182,183].

In addition, polyphenols possess the capacity to bind metals such as iron, aluminum, and copper [184]. This ion chelating capacity, although not desirable in deficient individuals, can be considered one of the reasons why polyphenols exert antidiarrheal properties [134], even if no specific actions have been confirmed with *E. coli* F4 so far.

Polyphenolic-rich extracts and their bioactive components are primarily recognized for their great antioxidant activity thanks to their hydroxyl groups, which can be oxidized by many molecules, including reactive oxygen species (ROS) [185]. The increased inflammation status of piglets caused by weaning leads to an increase in blood flow which produces a surge in the gastrointestinal ROS, a condition which has been coupled with an expansion of the *E. coli* gut population [186,187]. Moreover, as a response to inflammation, the production of NO provides a source of nitrogen that can be transformed into nitrate and used as a growth fuel for *E. coli* strains [188,189]. The detoxification of these reactive species by polyphenols could be an additional mechanism of action for their antimicrobial, antidiarrhoeic, and intestinal villi protective action against oxidative stress and morphological damages at weaning [190].

A study from Liu and colleagues compared the effect of hydrolysable tannins (HT) to pharmacological doses of ZnO in post-weaning piglets. The utilization of HT could reduce diarrhea incidence at the same extent of 2000 ppm of ZnO, and showed a synergistic tendency in the group supplemented with both HT and ZnO [191]. The same synergy was proved between HT and ZnO for diet digestibility, an effect probably exerted by the increased activity of enzymes such as trypsin, lipase, and α-amylase, the improvement of intestinal villi structure, and the enhanced antioxidative enzymes activity of the small intestine [191]. To the best of our knowledge, no other studies comparing polyphenol-rich extracts to pharmacological doses of ZnO or exploring their effects in piglets challenged with *E. coli* F4 are available, so further investigations will be required to elucidate their effectiveness as alternative strategies to manage PWD.

### 3.5. Prebiotics, Probiotics, and Symbiotics

Prebiotics are nondigestible feed ingredients that positively affect the host by stimulating the growth and/or the activity of a limited number of bacteria of the gastrointestinal microbiota [192]. A few studies have elucidated the advantages of supplementing different prebiotics to prevent damages exerted by the PWD main causative agent, *E. coli* F4. An in vitro study conducted on Caco-2 cells proved the ability of several milk-derived oligosaccharides fractions to avoid the adhesion of pathogenic *E. coli* to the cellular monolayer [193]. Selected galacto- and mannan-oligosaccharides reduce the in vitro adherence of *E. coli* F4 to intestinal mucins or porcine IPEC-J2 cells [194,195]. Moreover, orally-administered β-glucans decrease *E. coli* F4 colonization and diarrheic symptoms in post-weaning piglets [196]. The supplementation of an alginate-derived oligosaccharide could alleviate damages exerted by *E. coli* F4 infection by decreasing apoptosis and stimulating proliferation of enterocytes in challenged weanling piglets [197].

Probiotics are described as viable microorganisms that, if administered in sufficient amount, reach the intestine and exert positive effects for the host via direct inhibition of pathogens and/or stimulation of host defense systems [198]. The most common probiotics are lactic acid bacteria and yeasts. Roselli et al. demonstrated that in vitro addition of probiotic strains such as *Lactobacillus sobrius*, *Lactobacillus rhamnosus*, and *Bifidobacterium animalis* prevent *E. coli* F4 adhesion and tight-junction damages to intestinal epithelial cells, with a significant modulation of inflammatory cytokines [199,200]. Post-weaning piglets experimentally infected with *E. coli* F4 showed a significant reduction of diarrhea incidence when supplemented with *Lactobacillus rhamnosus* thanks to the modulation of intestinal microflora and inflammatory cytokines [201]. The addition of *Bacillus* species to weanling piglet diets after infection with *E. coli* F4 proved useful to reduce fecal score and increase the villi mitotic index through the enrichment of genes related to immune response [202]. A study by Nordeste and colleagues highlighted that probiotics beneficial effects are largely obtained by their ability to produce bioactive molecules: piglets challenged with *E. coli* F4 and supplemented with bioactive media conditioned with *Lactobacillus acidophilus* exhibited less PWD symptoms and better performance parameters because of an improvement in gut health and a lower *E. coli* F4 colonization [203]. Finally, feeding *Saccharomyces cerevisiae* in *E. coli* F4 infected piglets can lead to a reduction in diarrhea scores and duration, with a positive influence on animal growth capacity after weaning [204].

Some studies also explored the beneficial influences of combining both probiotics and prebiotics to improve the effective implantation and survival of health-promoting bacteria in the gastrointestinal tract [192]. The inclusion of a combination of 14% raw potato starch and *E. coli* probiotics could increase growth performance and reduce diarrhea incidence in piglets challenged with *E. coli* F4 [205]. A combination of *Lactobacillus plantarum* and lactulose proved effective to improve daily gain and intestinal morphology in post-weaning piglets orally challenged with *E. coli* F4, possibly through an increase in colonic trophic SCFA such as butyric acid [206]. In vitro administration of *Saccharomyces cerevisiae* and β-galactomannan oligosaccharides on porcine intestinal epithelial cells co-cultured with dendritic cells reduced the expression of ETEC-induced pro-inflammatory cytokines [207]. Moreover, the addition of *Lactobacillus rhamnosus* and fructooligosaccharides to *E. coli* F4 cultures could inactivate ETEC pathogenicity by reducing LT toxin expression [208].

Prebiotics and probiotics might represent an interesting approach to manage PWD and *E. coli* F4, thanks to their ability to secrete antibacterial molecules, inhibit pathogen virulence, compete for adherence sites, and modulate the animal immune system. However, the lack of consensus about the composition of the healthy piglet microbiota and the considerable differences between the attempted approaches still require further investigations [187].

### 3.6. Others

Researchers are always working on finding pioneering strategies against many diseases, including PWD and its etiological agents. The outcomes of several in vitro and in vivo trials provide promising results that could suggest other supplementary approaches to replace ZnO or against ETEC strains responsible of PWD. However, some of the alternatives which are being investigated remain at a research level, and have still not found a sustainable high-scale application and/or the approval of regulatory authorities.

#### 3.6.1. Antimicrobial Peptides

All the organisms naturally produce antimicrobial peptides (AMP), a heterogeneous class of small molecules that play a role in innate immunity providing immediate defense against bacterial, fungal, viral, and parasitic infections [138]. Moreover, it is suggested that they exert a low bacterial resistance acquisition [209]. In weanling piglets, other than directly targeting bacteria through many mechanisms of action, AMP can also modulate the immune system, improve growth performance, ensure an optimal intestinal structure, and suppress microbiota microorganisms such as harmful *E. coli* and *Salmonella* strains [137]. Even if no studies compared AMP to pharmacological doses of ZnO, a few research groups investigated the effects of two AMP in piglets challenged with ETEC. Wu et al. demonstrated that the supplementation of cecropin AD can improve intestinal morphology, reduce the count of *E. coli*, and increase the *Lactobacillus* count in the digesta, with a restoration of piglet growth parameters at the same extent of the group supplemented with colistin [210]. Some commensal *E. coli* strain can produce colicin, an AMP effective against pathogenic *E. coli*; Cutler and colleagues proved that its addition in diets of weanling piglet challenged with *E. coli* decreases PWD incidence and reduces the expression of pro-inflammatory cytokines [211]. Finally, it has been demonstrated that the in vitro overexpression of the BPI antimicrobial peptide can help in reducing ETEC adhesion to target cells and modulating the expression of several inflammatory cytokines [212].

#### 3.6.2. Bacteriophages

Bacteriophages are a large group of viruses that infects and replicates within bacterial cells. With the global concern generated by antimicrobial resistance, bacteriophages represent an interesting alternative against bacterial pathogens [213]. The utilization of bacteriophages active against *E. coli* F4 resulted in lower onset of diarrheic symptoms and reduced ETEC count in feces [214]. Moreover, bacteriophage supplementation ensured better growth parameters, fecal consistency score, villi:crypt ratio, and goblet cell density in *E. coli* F4 experimentally challenged weanling piglets [215]. To overcome their narrow spectrum of activity, some studies have explored with success the administration of a combination of more phages, with a significant improvement of many parameters, without affecting the total *E. coli* population of the gut microflora [216]. However, bacteriophages can elicit an immune response in the animal body, together with the possibility to induce bacterial resistance [217]. Finally, it has also been demonstrated that bacteriophages can carry antibiotic resistance genes, thereby representing dangerous horizontal gene transfer vectors [218].

#### 3.6.3. Egg Yolk Antibodies and Spray-Dried Plasma

Laying hens have attracted attention because of their capacity, after immunization, to concentrate specific antibodies (IgY) into the egg yolk, from which the IgY can be isolated to treat enteric pathogens [139]. In piglets experimentally challenged with *E. coli* F4, egg yolk antibodies significantly reduced the incidence of diarrhea, ensuring a positive weight gain and maximum survival [219]. The proposed mechanism of action against *E. coli* F4 is probably related to their ability to inhibit bacterial adhesion to mucus and intestinal cells [220,221]. However, no recent studies employing egg yolk antibodies are currently available, probably because of huge variabilities in IgY research studies due to their stability issues in the gastrointestinal tract and their cost of production [220,222].

Another immunoglobulin-related approach is represented by spray-dried plasma (SDP), a biologically active product obtained by industrial fractionation of animal blood. Studies have proved its effectiveness to control gastrointestinal infections, probably thanks to the direct activity of its immunoglobulin-rich fraction against pathogens or its supporting function for the immune system [140]. In post-weaning piglets orally challenged with *E. coli* F4, SDP supplementation ensured good levels of feed intake and weight gain [223], improved scours score and *E. coli* shedding in feces [224], provided better villi height:crypt depth ratio in the small intestine [225], and reduced the expression of inflammatory cytokines such as IL-8 and TNF-α [226]. However, the high production costs and the risks related to the potential presence of pathogens in SDP are endangering its effective implementation as a ZnO alternative strategy to prevent PWD [227].

## 4. Conclusions

Despite recent advances, the worldwide pig industry is still suffering from PWD and its main etiological agent, *E. coli* F4. At weaning, ZnO represents one of the major strategies for managing detrimental symptoms and bacterial infections, but severe threats arise from ZnO environmental pollution and its contribution to the spread of bacterial resistance. For a future free from medicinal ZnO doses, novel alternatives are necessary against pig related pathogens.

Numerous approaches have been attempted to treat PWD, but the proposed alternatives all show a significant gap with respect to ZnO’s efficacy. Moreover, the majority of all the analyzed studies lack a meticulous comparison between beneficial effects of pharmacological levels of ZnO and any of the newly-proposed methods, so it is often difficult to understand the real therapeutic or preventive actions of novel tools against PWD. The complexity in finding a unique effective replacement to ZnO probably lies in the supposedly multi-factorial and multi-target action of ZnO itself. Finding innovative alternatives to pharmacological ZnO for weaning pigs will probably require the combination of several of the strategies and tools presented across this review, to complement their different modes of action and targets synergistically.

In conclusion, the establishment of a novel approach against PWD will demand broad-spectrum interventions. Not only is it of crucial importance to find the optimal method to control growth, pathogenicity, and the virulence of PWD’s main etiological agent—*E. coli* F4—across all the pathogenic stages, but we must also figure out the best procedures to manage the weaning phase for piglets with pioneering environmentally-friendly molecules other than ZnO, along with the association of animal welfare with biosafety and nutritional measures, thereby adopting a holistic strategy.

## Figures and Tables

**Table 1:** Advantages and disadvantages of the main ZnO feeding alternatives for managing post-weaning diarrhea (PWD) and *Escherichia coli* F4 (K88) infections in weanling piglets. For each parameter, the up arrow “↑” means an increase, while the down arrow “↓” identifies a reduction.

ZnO Feeding Alternatives	Advantages	Disadvantages	References
Low protein diets	↓ proteolytic bacteria population ↓ pathogenic *E. coli* ↓ PWD symptoms ↓ pro-inflammatory cytokines	↓ pig productivity	[124,125]
High fiber diets	↓ PWD symptoms ↓ *E. coli* shedding ↓ *E. coli* adhesion ↑ SCFA production in digesta ↓ retention time of digesta	Few comparisons with pharmacological ZnO	[126,127,128]
Organic acids	Powerful antimicrobial activity Potential complete pharmacological Zn elimination ↑ gastric acidity ↑ nutrient digestibility ↑ growth performance ↓ PWD symptoms ↓ harmful coliforms ↓ inflammation ↑ intestinal morphology	Lack of comparisons with pharmacological ZnO Different activities between acids	[129,130]
Essential oils and nature identical compounds	Powerful antimicrobial, antioxidant, and anti-inflammatory activity ↑ growth performance ↑ diet digestibility ↓ PWD symptoms ↓ harmful coliforms ↓ bacterial virulence gene expression ↑ intestinal morphology	High variability in efficacy among different EO and molecules	[131,132,133]
Polyphenol-rich extracts	Considerable antimicrobial activity Ion chelating capacity High antioxidant activity ↓ PWD symptoms ↓ bacterial virulence gene expression ↓ bacterial adhesion to enterocytes ↓ bacterial toxin action ↑ intestinal morphology ↑ digestive enzymes activity	Few studies comparing polyphenols to pharmacological ZnO Polyphenols mechanism of action not yet fully elucidated	[134,135,136]
Antimicrobial peptides	Powerful antimicrobial activity ↓ bacterial resistance acquisition ↓ bacterial adhesion ↑ growth performance ↓ PWD symptoms ↑ intestinal morphology ↓ harmful coliforms ↑ immune response parameters	Lack of comparisons with pharmacological ZnO Need to investigate AMP pharmacokinetics	[137,138]
Egg yolk antibodies	↑ growth performance ↓ PWD symptoms ↓ bacterial adhesion to enterocytes	Lack of comparisons with pharmacological ZnO High cost Stability issues in the gastrointestinal tract	[139]
Spray-dried plasma	↑ growth performance ↓ PWD symptoms ↓ *E. coli* shedding ↑ intestinal morphology ↓ pro-inflammatory cytokines	Lack of comparisons with pharmacological ZnO High cost Complex production Potential presence of pathogens in SDP	[140]

## Data Availability

Not applicable.
